# The linear association between pulse pressure and diabetic retinopathy: a cross-sectional study

**DOI:** 10.3389/fcvm.2026.1918226

**Published:** 2026-09-30

**Authors:** Jinli He, Juan Ling, Xinlin Chen, Lina Zhang

**Affiliations:** 1 Department of Cardiology, Gansu Hospital of Traditional Chinese Medicine, Lanzhou, China; 2 Clinical College of Chinese Medicine, Gansu University of Chinese Medicine, Gansu Province, Lanzhou, China; 3 Department of Infection Management, The Gansu Provincial Hospital, Lanzhou, China; 4 Department of Geriatrics, Union Hospital, Tongji Medical College, Huazhong University of Science and Technology, Wuhan, China; 5 Department of Epidemiology and Biostatistics, Empower U, X&Y Solutions Inc., Boston, MA, United States; 6 Department of Critical Care Medicine, Gansu Hospital of Traditional Chinese Medicine, Lanzhou, China

**Keywords:** association, cross-sectional study, diabetic retinopathy, pulse pressure, type 2 diabetes mellitus

## Abstract

**Objective:**

This study aimed to evaluate the relationship between pulse pressure (PP) and the prevalence of diabetic retinopathy (DR) in individuals diagnosed with type 2 diabetes mellitus.

**Methods:**

This research is a secondary analysis of a cross-sectional study conducted between April 2002 and November 2004, which included 2,001 adults with diabetes mellitus (859 men and 1,142 women, mean age 64.1 ± 11.3 years) recruited from outpatient clinics in southern Taiwan. Comprehensive demographic and clinical data were collected, defining pulse pressure as the difference between systolic blood pressure (SBP) and diastolic blood pressure (DBP) measured in mmHg. The association between PP and DR was assessed using multivariable logistic regression, adjusting for potential confounders, and a generalized additive model (GAM) was utilized to explore non-linear relationships.

**Results:**

Elevated PP levels were consistently associated with higher odds of prevalent DR across progressively adjusted models. When PP was modeled per 10-mmHg increment, the association remained significant in Model 1 (OR 1.16; 95% CI 1.09–1.23; *P* < 0.0001), Model 2 (OR 1.13; 95% CI 1.06–1.21; *P* = 0.0002), and Model 3 (OR 1.09; 95% CI 1.02–1.17; *P* = 0.0136). When analyzed by tertiles, the middle tertile (50–61 mmHg) showed significant associations in the unadjusted model (OR 1.31; 95% CI 1.04–1.66; *P* = 0.0218) that diminished upon full adjustment (OR 1.19; 95% CI 0.92–1.52; *P* = 0.1828). In contrast, the high tertile (62–136 mmHg) maintained a significant association after adjustments (Model 1: OR 1.82; 95% CI 1.44–2.29; *P* < 0.0001; Model 3: OR 1.47; 95% CI 1.13–1.91; *P* = 0.0039).

**Conclusions:**

This study indicates higher PP was associated with higher odds of prevalent DR in individuals with type 2 diabetes.

## 1 Introduction

Diabetic retinopathy (DR) is a major microvascular complication of diabetes mellitus and a leading cause of vision loss among working-age adults worldwide (1). It is characterized by progressive retinal microvascular damage, which manifests as microaneurysms, hemorrhages, capillary nonperfusion, and pathological neovascularization in advanced stages (2). The pathogenesis of DR is primarily driven by chronic hyperglycemia, leading to endothelial dysfunction, inflammation, and breakdown of the blood-retinal barrier, ultimately resulting in retinal ischemia and vision impairment (3–5).

Pulse pressure (PP), defined as the difference between systolic and diastolic blood pressure, reflects arterial stiffness and reduced vascular compliance (6). In patients with diabetes mellitus, increased arterial stiffness results in elevated PP, which can transmit excessive pulsatile stress to the microcirculation, including the retinal vasculature (7, 8). Emerging evidence has increasingly focused on the link between arterial stiffness, indicated by pulse pressure, and the development and progression of DR. Studies have reported that elevated pulse wave velocity and increased central and peripheral pulse pressures correlate with the severity of DR and its microvascular manifestations. For example, higher brachial-ankle PWV has been independently associated with more advanced stages of DR, suggesting that arterial stiffness may contribute to retinal microvascular damage (9, 10). Additionally, increased pulse pressure and its variability have been identified as significant risk factors for DR prevalence in patients with type 2 diabetes mellitus (11). Collectively, these findings suggest that systemic arterial stiffness, as reflected by elevated PP and its variability, may play an important role in the development and progression of diabetic retinopathy. However, important gaps remain regarding the shape of the PP–DR relationship (e.g., whether the association is linear or involves a threshold) and the extent to which the association is independent of key microvascular-related comorbidities and treatments. Therefore, in this cross-sectional study, we aimed to (1) quantify the association between PP and prevalent DR using multivariable logistic regression; (2) assess potential non-linearity and dose–response patterns using generalized additive models (GAM); (3) strengthen confounding control by additionally accounting for renal indices (creatinine, eGFR, and UACR) and major antihypertensive medication classes (ACEI/ARBs, β-blockers, and calcium channel blockers); and (4) examine robustness across a broad range of clinically relevant subgroups.

## 2 Methods

### 2.1 Study population

We undertook a secondary analysis of an existing cross-sectional dataset originally published by Chen et al. (12) in PLOS ONE (DOI: 10.1371/journal.pone.0134718). This dataset is publicly accessible under a licensing agreement that allows for unrestricted academic reuse. The source data were collected from April 2002 to November 2004. The present study is a secondary analysis conducted using this publicly available, de-identified dataset. Although the data were collected in an earlier period, we performed the current analysis because the dataset contains the key exposure (pulse pressure), outcome (diabetic retinopathy assessed by standardized ophthalmic examinations), and relevant covariates needed to evaluate the PP-DR association using updated multivariable and non-linear modeling approaches. Importantly, the biological relationship between arterial stiffness-related hemodynamic load, as reflected by PP, and retinal microvascular damage is unlikely to be time-dependent, and PP remains a routinely measured clinical variable; diabetic retinopathy also remains a leading cause of visual impairment despite advances in diabetes care. Nevertheless, because the management of diabetes mellitus and retinopathy screening have evolved considerably since 2002–2004 (e.g., more intensive glycemic control, wider use of renin–angiotensin system blockers and lipid-lowering therapy, and modern retinal imaging and screening programs), both the distribution of PP and the case-mix of DR may differ in contemporary populations. The potential impact of these changes on the generalizability of our findings is addressed in the Limitations.

Eligibility and definitions followed the parent study (Chen et al.) (12) and the original dataset documentation. Diagnosis was established through one of the following criteria: (1) fasting plasma glucose levels equal to or exceeding 7.0 mmol/L (126 mg/dL); (2) 2-hour plasma glucose levels reaching or surpassing 11.1 mmol/L (200 mg/dL) during a 75-g oral glucose tolerance test; (3) glycated hemoglobin (HbA1c) levels of 6.5% (48 mmol/mol) or greater; or (4) in individuals exhibiting classic symptoms of hyperglycemia or experiencing a hyperglycemic crisis, a random plasma glucose level of 11.1 mmol/L (200 mg/dL) or more. In cases where hyperglycemia was not definitively present, the results for criteria (1)–(3) necessitated confirmation through repeat testing on a separate occasion. In the parent study, patients with type 1 diabetes mellitus [defined by the presence of diabetic ketoacidosis, acute hyperglycemic symptoms accompanied by significant ketonuria (≥3+), or the requirement for continuous insulin administration within one year of diagnosis] were excluded, as were patients receiving dialysis, those with an estimated glomerular filtration rate <15 mL/min/1.73 m^2^, and those who had undergone renal transplantation. After these exclusions, the final analytic cohort comprised 2,001 participants (mean age 64.1 ± 11.3 years), including 859 men and 1,142 women. A flow diagram of the participant selection process is shown in Figure 1. The design and reporting of this study adhered to the STROBE guidelines for cross-sectional research.

**Figure 1:**
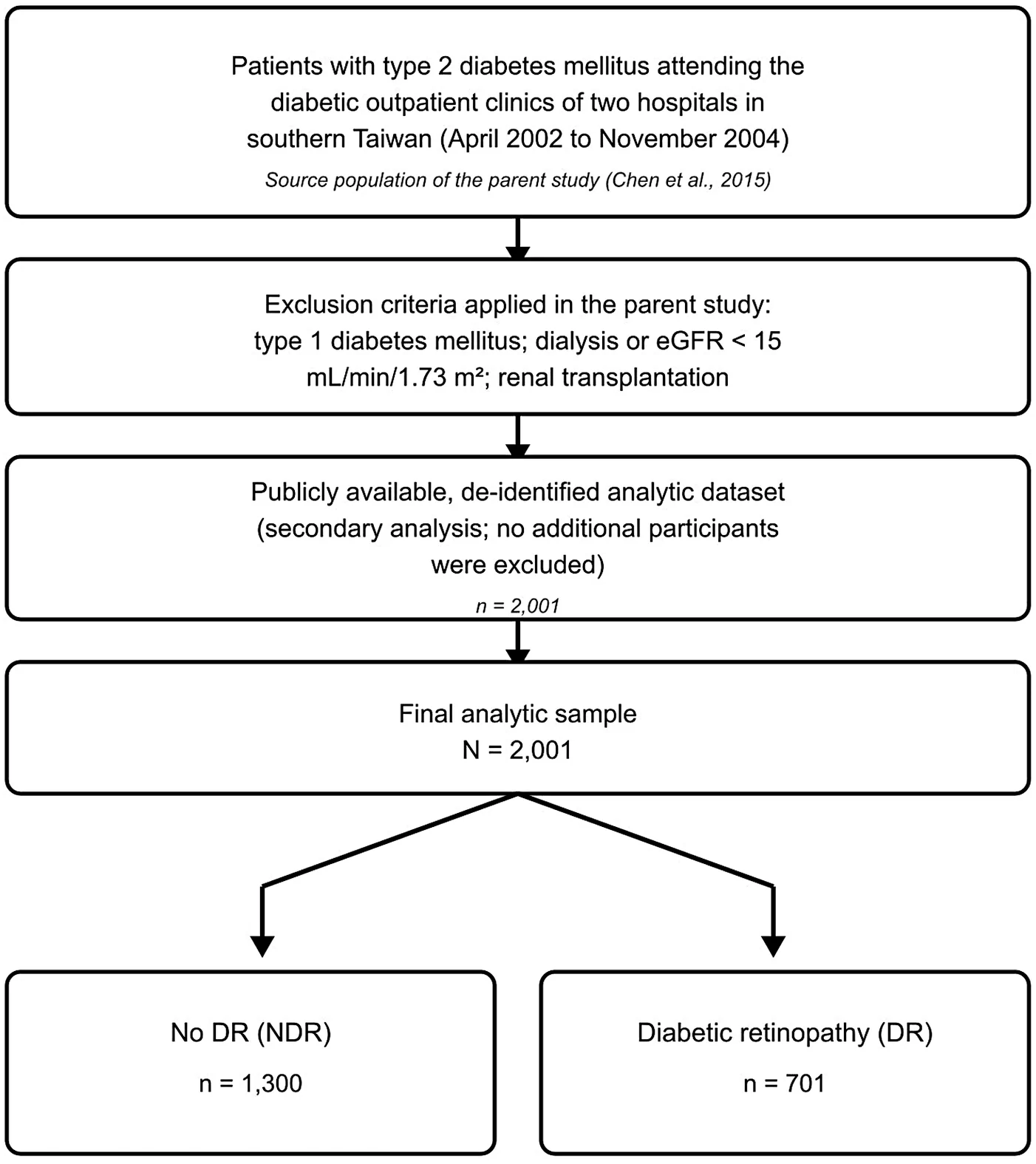
Flow diagram of participant selection. DR, diabetic retinopathy; eGFR, estimated glomerular filtration rate; NDR, no diabetic retinopathy.

### 2.2 Ethics statement

The anonymized dataset utilized in this secondary analysis was obtained from the research conducted by Chen et al. (12). The protocol for the primary study received thorough evaluation and approval from the Institutional Review Board at Kaohsiung Medical University Hospital (approval No. KMUHIRB-E-20150029) and was executed in compliance with the Declaration of Helsinki, pertinent international ethical standards, and applicable local regulations. Informed consent, in written form, was obtained from all participants, which encompassed their agreement to partake in the study and the dissemination of de-identified clinical data. Given that the current investigation focused on secondary analysis of a publicly accessible, de-identified dataset and did not involve any new participant recruitment or intervention, further ethical approval was deemed unnecessary.

### 2.3 Variables

Demographic and clinical data, including age, sex, and comorbidities, were extracted from medical records and standardized patient interviews. Anthropometric measurements were taken using a calibrated digital scale and stadiometer; participants were instructed to stand barefoot with their heels together and their backs aligned against the stadiometer. Weight was documented to the nearest 0.1 kg, while height was recorded to the nearest 0.1 cm. The body mass index (BMI) was calculated by dividing weight (in kg) by the square of height (in m²). Blood pressure was measured using an automated oscillometric device after at least 5 min of seated rest. In the parent study, the VP-1000 device automatically and simultaneously measured blood pressures in both arms. SBP and DBP used in the present secondary analysis were obtained from the parent dataset as recorded/derived under the original protocol. Hypertension guidelines recommend measuring BP in both arms at the initial assessment and using the arm with the higher systolic BP for subsequent measurements when a consistent inter-arm difference is present (13, 14). Pulse pressure (PP) was calculated as the difference between systolic and diastolic blood pressure (in mmHg). Because PP is mathematically derived from SBP and DBP, SBP and DBP were not included as covariates in models where PP was the exposure, to avoid overadjustment and collinearity and to improve interpretability. The ankle-brachial index (ABI) was determined as the ratio of ankle to brachial systolic pressure. An abnormal (pathological) ABI was defined as ABI < 0.90 or ABI ≥ 1.30 in either leg, consistent with the parent study (15, 16). According to the World Health Organization (WHO), stroke is defined as a clinical syndrome characterized by the abrupt onset of focal neurological deficits that persist for over 24 h or lead to death, with no identifiable cause aside from one of vascular origin (17).

### 2.4 DR confirmation

DR was assessed by board-certified ophthalmologists using dilated color fundus photography and a standardized grading system. The presence and severity of DR were graded using the International Clinical Diabetic Retinopathy Disease Severity Scale, consistent with the parent study (18). Because the present study is a secondary analysis of the parent-study dataset (12), the participant-level DR classification recorded in the de-identified dataset was used for all analyses. In the parent study, DR was evaluated by experienced ophthalmologists after pupillary dilation, with fluorescein angiography performed when needed, and DR was classified as no DR, non-proliferative DR (NPDR), or proliferative DR (PDR) (12). Eye-specific details of the grading (e.g., whether both eyes were examined and how discrepancies between eyes were handled) are not described in the parent-study publication or in the dataset documentation available for this secondary analysis; we therefore did not make assumptions about eye-level grading. For the primary analysis, NPDR and PDR were combined into a single category representing any (prevalent) DR (present vs. absent); separate analyses of NPDR and PDR as distinct outcomes were not performed.

### 2.5 Statistical analysis

Categorical variables were expressed as counts and percentages. Continuous variables were presented as mean ± standard deviation for normally distributed variables and as median (interquartile range, 25th–75th percentiles) for non-normally distributed variables. Generalized additive models (GAMs) were used to obtain smooth curves for visualizing the exposure-response relationship between PP and prevalent DR. To formally test whether the association between PP and prevalent DR deviates from linearity, we compared a single-line logistic model with a two-piecewise (threshold) logistic model using a log-likelihood ratio test; the resulting *P* value (P for non-linearity) is reported in the Results. To facilitate clinical interpretation, the continuous exposure was modeled per 10-mmHg increment (i.e., PP/10), and the corresponding odds ratios are reported per 10 mmHg.

Prespecified subgroup and interaction analyses were performed according to sex, age tertiles, body mass index (BMI) tertiles, lipid parameters (LDL-C, HDL-C, and triglycerides), renal function indices [serum creatinine, estimated glomerular filtration rate [eGFR], and urinary albumin-to-creatinine ratio [UACR] > 30 mg/g], ankle–brachial index (ABI) tertiles, ischemic heart disease status, and use of antihypertensive medications (ACE inhibitors/ARBs, β-blockers, and calcium channel blockers). Potential multicollinearity among independent variables was assessed using variance inflation factors (VIFs). A VIF ≥ 5 was considered indicative of substantial multicollinearity. VIF diagnostics were performed during model building to ensure the stability of the multivariable regression models (19, 20). This procedure allowed for the retention of key variables closely related to the study outcomes, thereby improving the overall model performance and interpretability. All statistical analyses were conducted using EmpowerStats software (version 4.1; X&Y Solutions, Inc., Boston, MA, USA) and R software (version 4.2.0; The R Foundation for Statistical Computing, Vienna, Austria). The covariates included in the fully adjusted model (Model 3) were selected *a priori* as established correlates of PP and/or DR along the hypothesized atherosclerosis–microvascular pathway, including demographic and metabolic variables (age, sex, BMI, triglycerides, LDL-C, HDL-C, HbA1c), renal indices (creatinine, eGFR, UACR > 30 mg/g), peripheral arterial status (ABI), ischemic heart disease, and antihypertensive medication classes (ACEI/ARB, β-blockers, and calcium channel blockers). We acknowledge that some of these variables—particularly antihypertensive treatment and renal/peripheral vascular indices—may lie on the causal pathway between elevated PP and DR, and that adjusting for such intermediates could attenuate the association (potential over-adjustment). To allow the impact of successive adjustment to be evaluated directly, minimally (Model 1), partially (Model 2), and fully adjusted (Model 3) models are presented. Standardized differences were reported descriptively and were not used for hypothesis testing. For continuous variables, the standardized difference was calculated as |mean_1_—mean_2_|/√[(SD_1_^2^ + SD_2_^2^)/2], where SD_1_ and SD_2_ are the group-specific standard deviations. For the binary categorical variables in Table 1, it was calculated as |p_1 −_ p_2_|/√{[p_1_(1_ −_ p_1_) + p_2_(1_ −_ p_2_)]/2}, where p_1_ and p_2_ are the group-specific proportions. The 95% confidence interval was calculated as d ± 1.96 × SE, where d is the standardized difference and SE = √[(n_1_ + n_2_)/(n_1_ × n_2_) + d^2^/[2(n_1_ + n_2_)]]; n_1_ and n_2_ are the group-specific sample sizes. These confidence intervals were based on a large-sample normal approximation.

**Table 1:** Baseline characteristics of the study participants between two groups (*n* = 2,001).

Characteristic	No retinopathy (*N* = 1,300)	Retinopathy (*N* = 701)	Standardized difference	*P*-value
Age (years)	63.33 ± 11.83	65.40 ± 10.21	0.19 (0.10, 0.28)	<0.001
BMI (kg/m²)	25.80 ± 3.64	25.91 ± 3.44	0.03 (−0.06, 0.12)	0.505
SBP (mmHg)	133.71 ± 18.37	137.07 ± 19.27	0.18 (0.09, 0.27)	<0.001
DBP (mmHg)	77.84 ± 11.07	77.72 ± 11.71	0.01 (−0.08, 0.10)	0.831
Pulse Pressure (mmHg)	55.87 ± 14.89	59.35 ± 15.74	0.23 (0.13, 0.32)	<0.001
Cholesterol (mg/dL)	186.53 ± 38.29	184.12 ± 39.28	0.06 (−0.03, 0.15)	0.184
Triglycerides (mg/dL)	124.0 (89.2–181.0)	129.0 (93.0–174.8)	0.0 (−0.1, 0.1)	0.995
HbA1c (%)	7.6 ± 1.7	7.8 ± 1.6	0.1 (0.0, 0.2)	0.023
LDL-C (mg/dL)	104.80 ± 28.52	103.37 ± 28.43	0.05 (−0.04, 0.14)	0.284
HDL-C (mg/dL)	50.11 ± 13.23	48.45 ± 12.67	0.13 (0.04, 0.22)	0.007
Creatinine (mg/dL)	1.05 ± 0.36	1.13 ± 0.42	0.22 (0.12, 0.31)	<0.001
eGFR (mL/min/1.73 m^2^)	70.45 ± 19.37	65.42 ± 19.84	0.26 (0.16, 0.35)	<0.001
ABI	1.10 ± 0.10	1.10 ± 0.12	0.04 (−0.05, 0.14)	0.346
Sex			0.03 (−0.06, 0.12)	0.565
Male	552 (42.46%)	307 (43.79%)		
Female	748 (57.54%)	394 (56.21%)		
UACR > 30 mg/g			0.30 (0.20, 0.39)	<0.001
No	911 (70.08%)	392 (55.92%)		
Yes	389 (29.92%)	309 (44.08%)		
Ischemic Heart Disease			0.12 (0.03, 0.21)	0.009
No	1,102 (84.77%)	562 (80.17%)		
Yes	198 (15.23%)	139 (19.83%)		
β-blocker use (%)			0.07 (−0.02, 0.17)	0.11
No	1,006 (77.74%)	522 (74.57%)		
Yes	288 (22.26%)	178 (25.43%)		
ACEI and/or ARB use (%)			0.20 (0.11, 0.29)	<0.001
No	381 (29.44%)	146 (20.86%)		
Yes	913 (70.56%)	554 (79.14%)		
Calcium channel blocker use (%)			0.27 (0.18, 0.37)	<0.001
No	848 (65.53%)	365 (52.14%)		
Yes	446 (34.47%)	335 (47.86%)		
Stroke				
No	1,249 (96.1%)	653 (93.2%)	0.1 (0.0, 0.2)	0.004
Yes	51 (3.9%)	48 (6.8%)		

ABI: ankle-brachial index; UACR: urinary albumin-to-creatinine ratio; BMI, body mass index; SBP, systolic blood pressure; DBP, diastolic blood pressure; eGFR: estimated glomerular filtration rate; LDL-C, low-density lipoprotein cholesterol; HDL-C, high-density lipoprotein cholesterol; ABI, ankle-brachial index (unitless); ACEI, angiotensin-converting enzyme inhibitor; ARB, angiotensin II receptor blocker.

## 3 Results

A total of 2,001 individuals with type 2 diabetes mellitus were included in the study. The mean age of the overall study population was 64.1 ± 11.3 years. Baseline characteristics for the NDR and DR groups are summarized in Table 1. Standardized differences were used to quantify between-group imbalance independent of sample size; as a rule of thumb, an absolute standardized difference <0.10 indicates negligible imbalance, whereas ≥0.10 suggests a potentially meaningful difference. Standardized differences were reported alongside *P* values because the two measures provide complementary information: *P* values test the null hypothesis of no between-group difference and may be influenced by sample size, whereas standardized differences quantify the magnitude of between-group imbalance on a unit-free scale that does not directly depend on sample size.

Table 2 summarizes the association between PP and DR. When modeled as a continuous variable per 10-mmHg increment, higher PP was associated with higher odds of DR across progressively adjusted models: Model 1 (OR 1.16; 95% CI 1.09–1.23; *P* < 0.0001), Model 2 (OR 1.13; 95% CI 1.06–1.21; *P* = 0.0002), and Model 3 (OR 1.09; 95% CI 1.02–1.17; *P* = 0.0136). A formal likelihood-ratio test comparing a single-line model with a two-piecewise (threshold) model indicated no significant departure from linearity (P for non-linearity = 0.367); although a breakpoint was identified at 45 mmHg, the difference in slopes between the two segments was not statistically significant (*P* = 0.363). In tertile analyses using the low tertile (6–49 mmHg) as reference, the middle tertile (50–61 mmHg) showed an association in the unadjusted model (OR 1.31; 95% CI 1.04–1.66; *P* = 0.0218) that attenuated and became non-significant after full adjustment (OR 1.19; 95% CI 0.92–1.52; *P* = 0.1828). By contrast, the high tertile (62–136 mmHg) exhibited consistently elevated odds that persisted after adjustment—Model 1 (OR 1.82; 95% CI 1.44–2.29; *P* < 0.0001), Model 2 (OR 1.67; 95% CI 1.30–2.14; *P* < 0.0001), and Model 3 (OR 1.47; 95% CI 1.13–1.91; *P* = 0.0039)—indicating a graded, independent association concentrated at higher PP levels. No substantial multicollinearity was observed in the fully adjusted models, with all covariates showing VIF < 5.

**Table 2:** The association between PP and DR.

Pulse Pressure (mmHg)	Model 1	Model 2	Model 3
	[OR (95% CI), P]	[OR (95% CI), P]	[OR (95% CI), P]
Overall (per 10 mmHg)	1.16 (1.09, 1.23) < 0.0001	1.13 (1.06, 1.21) 0.0002	1.09 (1.02, 1.17) 0.0136
Pulse Pressure (mmHg) tertile			
Low (6–49 mmHg)	1	1	1
Middle (50–61 mmHg)	1.31 (1.04, 1.66) 0.0218	1.24 (0.97, 1.58) 0.0837	1.19 (0.92, 1.52) 0.1828
High (62–136 mmHg)	1.82 (1.44, 2.29) < 0.0001	1.67 (1.30, 2.14) < 0.0001	1.47 (1.13, 1.91) 0.0039

For the continuous PP model, ORs are reported per 10-mmHg increase in PP. Model 1: no covariates were adjusted; Model 2: Sex, age; Model 3: Sex, age, BMI, triglycerides, LDL-C, HDL-C, HbA1c, Creatinine, eGFR, Ischemic Heart Disease, ACEI and/or ARB use, *β*-blocker use, UACR > 30 mg/g, Calcium channel blocker use and ABI were adjusted. Model 3 was based on 1,976 participants with complete covariate data, whereas Models 1 and 2 included 2,001 participants; missing covariate values were handled by complete-case analysis.

To further explore whether the association between PP and DR differed across clinical strata, we performed exploratory subgroup analyses and tested interactions (Figure 2). Given the number of subgroup strata and interaction tests, these analyses are subject to multiplicity and potential inflation of type I error; therefore, subgroup findings should be interpreted cautiously, particularly when confidence intervals are close to the null. Overall, all *P* values for interaction were >0.05 (minimum 0.096), and none remained significant after Bonferroni correction (threshold 0.0036 for the 14 subgroup variables), providing no clear evidence of effect modification across the examined subgroups. Because these subgroup analyses were exploratory and hypothesis-generating, no formal correction for multiple comparisons (e.g., Bonferroni adjustment) was applied; interaction *P* values are therefore reported without adjustment and should be interpreted descriptively. These exploratory results are presented solely to evaluate the consistency of the primary association across clinical strata and are not used for primary inference.

**Figure 2:**
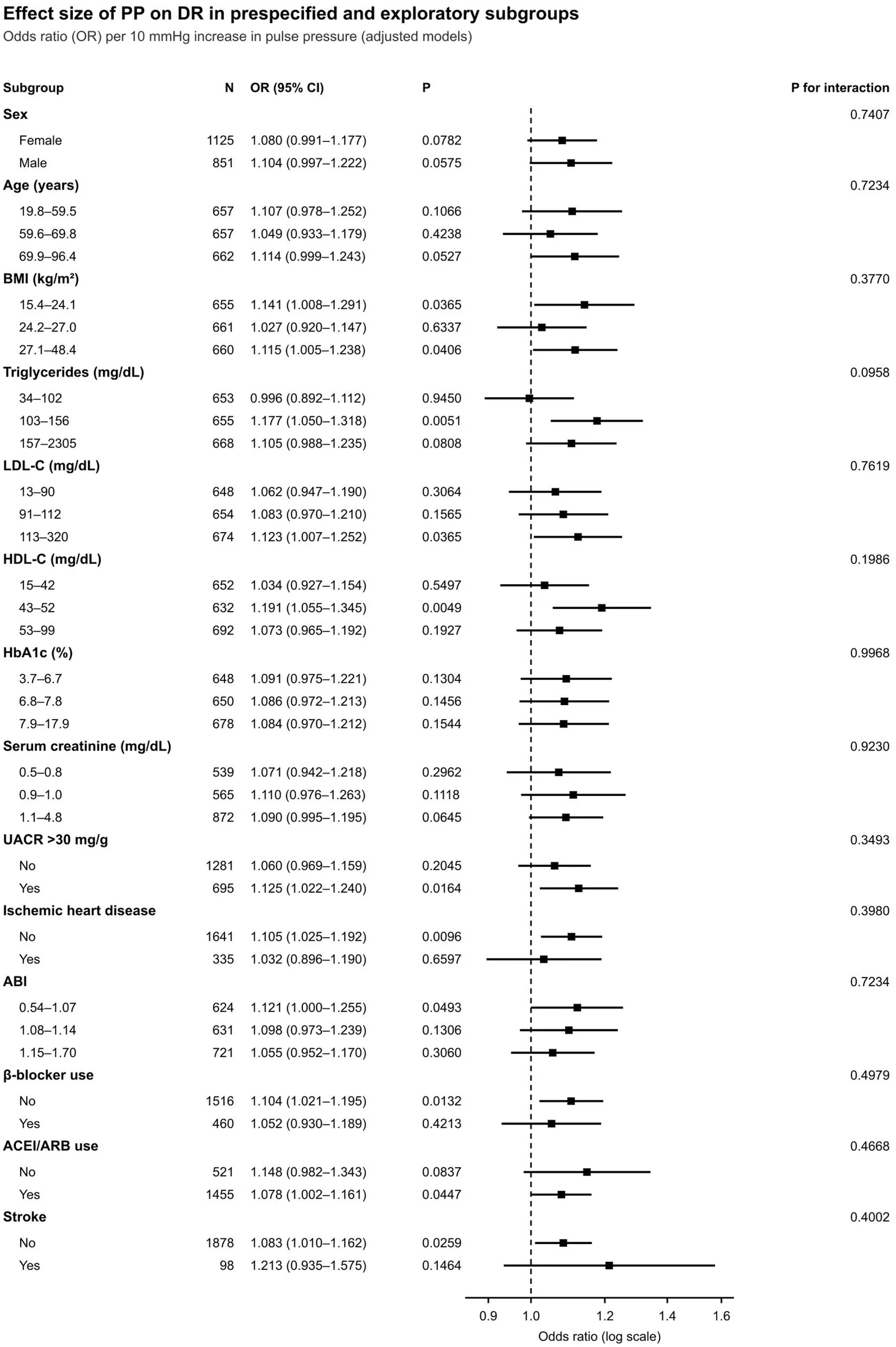
Effect size of PP on DR in prespecified and exploratory subgroups. Note 1: ORs are reported per 10-mmHg increase in PP. Sex, age, BMI, triglycerides, LDL-C, HDL-C, HbA1c, Creatinine, eGFR, Ischemic Heart Disease, ACEI and/or ARB use, β-blocker use, UACR > 30 mg/g, Calcium channel blocker use and ABI were adjusted. Note 2: Effect estimates for PP were derived from models including a PP × subgroup interaction term; all models were adjusted for the covariates listed in Note 1, and the *P* value for interaction is reported.

The application of smooth-spline fitting revealed a distinct, approximately linear positive association between PP and the prevalence of DR; specifically, elevated PP levels were associated with a higher probability of prevalent DR (Figure 3). Age-stratified evaluations substantiated this observation throughout all tertiles, demonstrating that the curves diverged at elevated PP levels, with the oldest tertile exhibiting the highest prevalence. This suggests that the association between PP and DR is consistent across different age groups and may be more pronounced in older populations (Figure 4). When analyzing within body mass index (BMI) strata, the overarching trend remained positive; however, a modest departure from a strictly linear pattern was observed within the middle BMI tertile. Furthermore, at equivalent PP levels, individuals in the highest BMI tertile tended to display the most substantial prevalence of DR, suggesting that BMI may modify the association between PP and DR (Figure 5). Additionally, across HbA1c tertiles, PP was positively associated with the probability of DR. Higher PP corresponded to a higher probability of DR, with a more pronounced association observed in the high HbA1c group (Figure 6).

**Figure 3:**
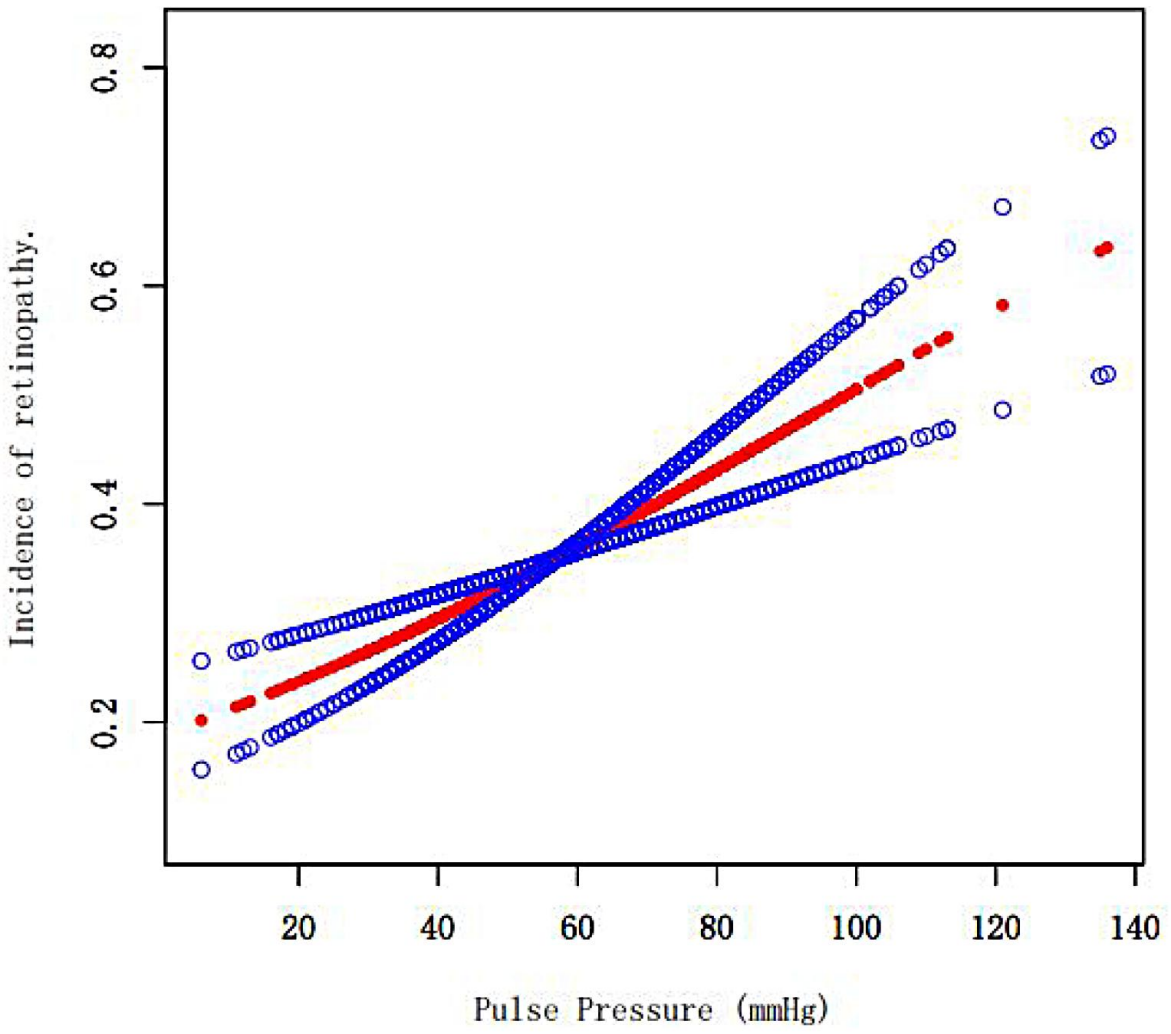
The association between PP and DR.

**Figure 4:**
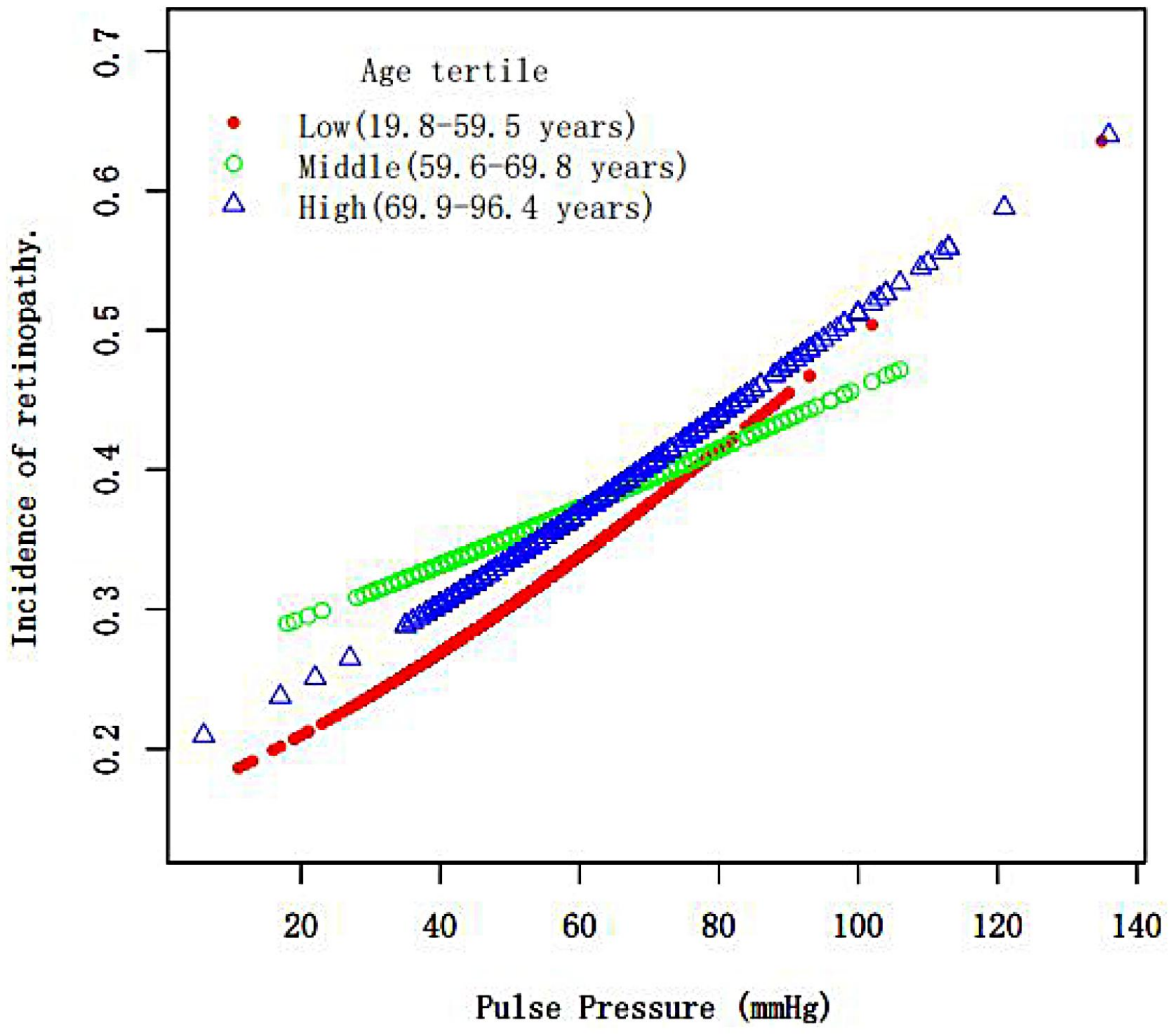
Association between PP and DR, stratified by age tertiles.

**Figure 5:**
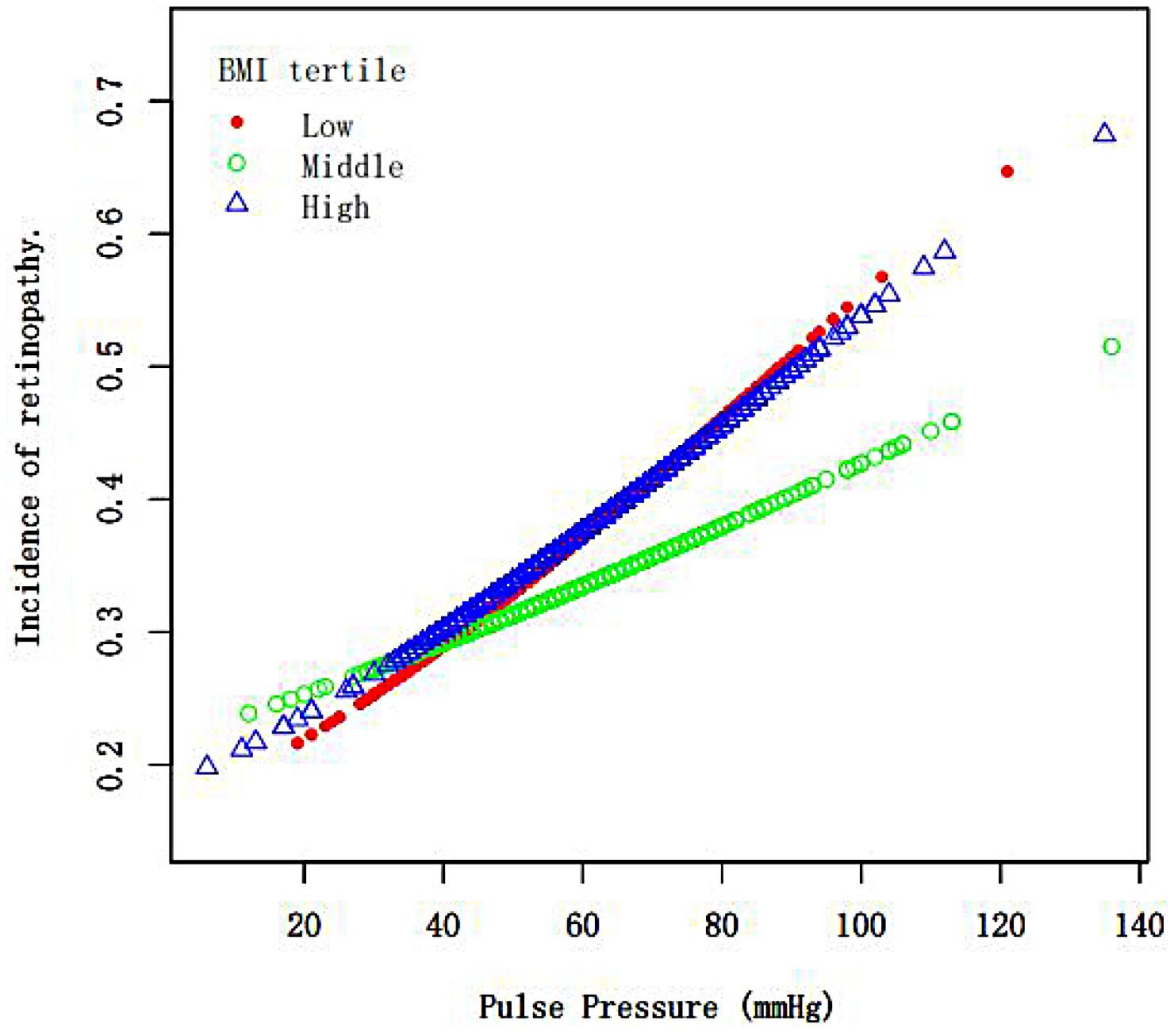
Association between PP and DR, stratified by BMI tertiles.

**Figure 6:**
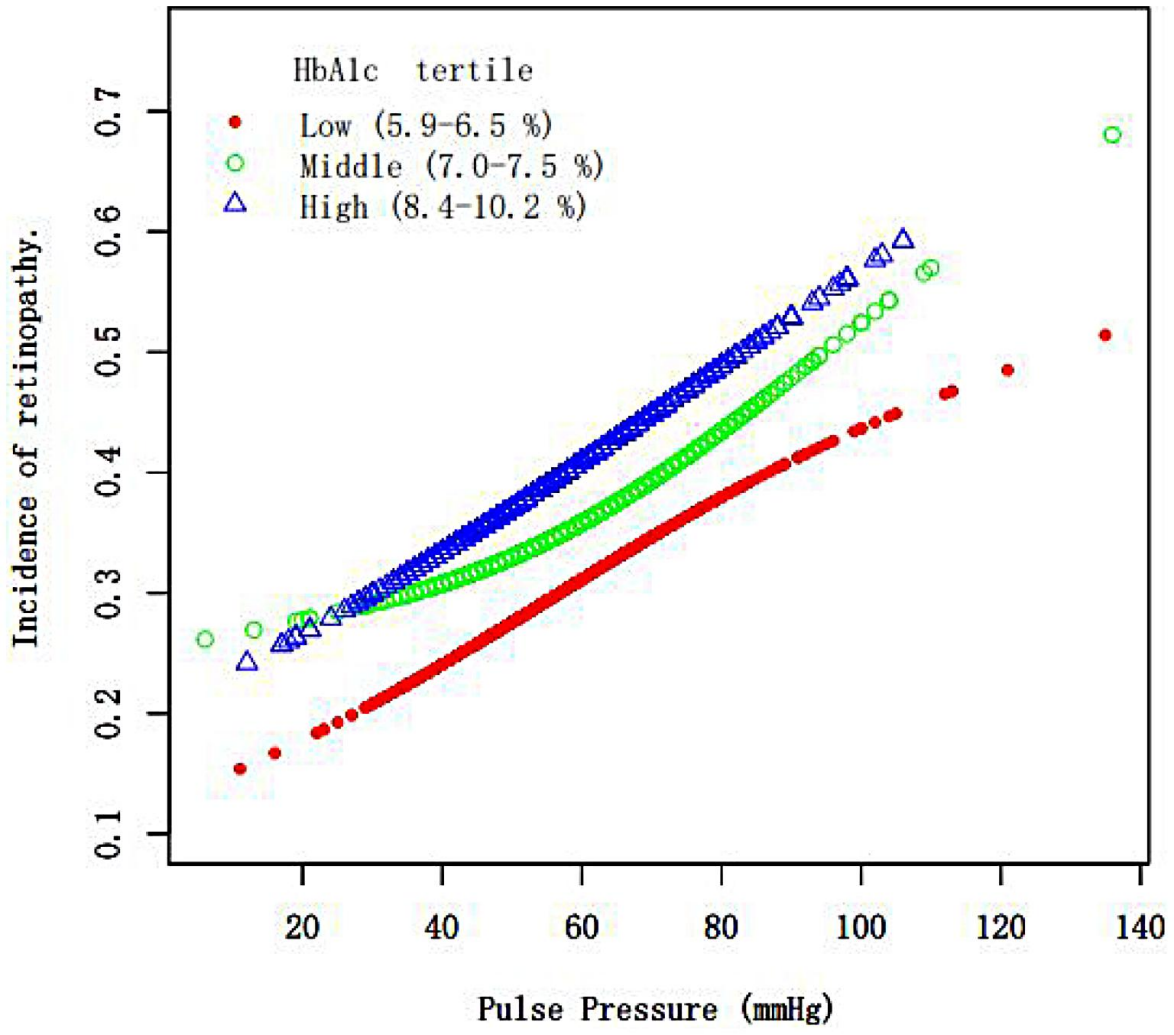
Association between PP and DR, stratified by HbA1c tertiles. Figure 2 shows the effect sizes of PP on the odds of prevalent DR in prespecified and exploratory subgroups (ORs are reported per 10-mmHg increase in PP). Figures 3–6 show GAM-derived smooth curves for the association between PP and the probability of prevalent DR: Figure 3, overall association; Figure 4, stratified by age tertiles; Figure 5, stratified by BMI tertiles; Figure 6, stratified by HbA1c tertiles. In Figures 3–6, the red line represents the estimated smooth curve and the blue bands represent the 95% confidence interval. Covariates were adjusted as in Figure 2, except for the stratification variable.

## 4 Discussion

In this cross-sectional study of 2,001 adults with type 2 diabetes mellitus, higher pulse pressure (PP) was associated with higher odds of prevalent diabetic retinopathy (DR). In the fully adjusted model, each 10-mmHg increment in PP was associated with an odds ratio (OR) of 1.09 (95% CI 1.02–1.17; *P* = 0.0136). Compared with the lowest PP tertile (6–49 mmHg), the highest tertile (62–136 mmHg) was associated with higher odds of DR (OR 1.47; 95% CI 1.13–1.91; *P* = 0.0039). The dose–response relationship was approximately linear across the observed PP range (Figure 3), and the subgroup and interaction analyses provided no evidence of effect modification by age, sex, BMI, or other clinical characteristics (Figure 2).

Our findings are consistent with previous studies that examined PP or closely related arterial-stiffness indices in relation to DR. Yamamoto et al. (21) reported that PP outperformed systolic blood pressure in predicting severe diabetic eye disease in a large nationwide cohort; Liu et al. (22) found that higher brachial–ankle pulse wave velocity was associated with more severe DR in patients with type 2 diabetes mellitus; and Lou et al. (11) showed that both mean PP and PP variability predicted incident DR. Our study adds to this evidence by formally characterizing the dose–response shape with GAM (an approximately linear pattern), by comprehensively adjusting for renal indices (creatinine, eGFR, UACR) and antihypertensive medication classes that are often incompletely addressed in earlier analyses, and by demonstrating consistent associations across clinically relevant subgroups. Differences in design and measurement—cross-sectional assessment of prevalent DR vs. time-to-event analyses, brachial PP vs. pulse wave velocity, and the absence of PP variability—may explain quantitative differences among studies. Mechanistically, elevated PP transmits excessive pulsatile energy to the retinal microvasculature and may promote endothelial dysfunction, oxidative stress, breakdown of the blood–retina barrier, and retinal ischemia, providing a biologically coherent explanation for the observed association. If confirmed in prospective studies, these findings support a vascular-stiffness–pulsatility pathway to DR and suggest that PP, a simple and inexpensive clinical measure, may help identify patients who could benefit from more intensive cardiovascular risk-factor management; interventional trials are nevertheless required to establish whether PP-targeted strategies confer microvascular benefits.

Several limitations should be considered. First, because this was a cross-sectional study, causal relationships and the temporal sequence between PP and DR cannot be established; accordingly, our findings should be interpreted as associations with prevalent DR, and PP should not be regarded as a screening or risk-prediction tool on the basis of this study. Second, information on the duration of diabetes mellitus—one of the strongest determinants of DR—was not available. Because unmeasured differences in diabetes duration could confound the association between PP and DR (longer diabetes duration is associated both with higher DR risk and with more advanced vascular disease), residual confounding related to long-term glycemic exposure cannot be excluded, and this limitation may have a substantial impact on the interpretation of the observed association. Third, because PP is mathematically derived from systolic and diastolic blood pressure (SBP and DBP), SBP and DBP were not simultaneously included in models in which PP was the exposure, to avoid multicollinearity; residual confounding related to overall blood pressure status may therefore remain. Fourth, some covariates in the fully adjusted model (e.g., renal indices, ABI, and antihypertensive medication classes) may lie on the causal pathway between elevated PP and DR, and adjustment for potential intermediates could attenuate rather than exaggerate the association; we therefore present progressively adjusted models (Models 1–3) so that the impact of successive adjustment can be directly appraised. Finally, the data were collected between 2002 and 2004 from two hospitals in southern Taiwan. Because the management of diabetes mellitus and the screening and treatment of DR have changed substantially since that period, the distribution of PP and the severity profile of DR in contemporary populations may differ. These temporal changes, together with the use of a historical, publicly available dataset, may limit direct generalizability, and our findings should be confirmed in contemporary cohorts.

## 5 Conclusion

Pulse pressure is independently and positively associated with the prevalence of DR among adults with type 2 diabetes mellitus. The association is approximately linear, with higher PP corresponding to higher odds of DR after adjustment for demographics, lipids, renal indices, peripheral vascular status, and antihypertensive classes. These findings suggest that PP is an easily obtainable clinical measure associated with prevalent DR. However, because of the cross-sectional design, the present results do not establish predictive validity or clinical benefit, and PP should not yet be recommended for clinical risk classification or DR screening. Prospective studies are needed to determine whether PP improves the prediction of incident DR or its progression, and whether PP-targeted interventions confer microvascular benefits.

## Data Availability

The original contributions presented in the study are included in the article/Supplementary Material, further inquiries can be directed to the corresponding author/s.
